# Effect of mechanistic/mammalian target of rapamycin complex 1 on mitochondrial dynamics during skeletal muscle hypertrophy

**DOI:** 10.14814/phy2.14789

**Published:** 2021-03-04

**Authors:** Kazuki Uemichi, Takanaga Shirai, Hideto Hanakita, Tohru Takemasa

**Affiliations:** ^1^ Graduate School of Comprehensive Human Sciences University of Tsukuba Tsukuba Japan; ^2^ Research Fellow of the Japan Society for the Promotion of Science Tokyo Japan; ^3^ Faculty of Health and Sports Sciences University of Tsukuba Tsukuba Japan

**Keywords:** mitochondrial dynamics, mTOR signaling, skeletal muscle hypertrophy

## Abstract

Mechanistic/mammalian target of rapamycin (mTOR) is a central factor of protein synthesis signaling and plays an important role in the resistance training‐induced increase in skeletal muscle mass and subsequent skeletal muscle hypertrophy response. In particular, mTOR complex 1 (mTORC1) promotes protein synthesis in ribosomes by activating the downstream effectors, p70S6K and 4EBP1, in skeletal muscle and is highly sensitive to rapamycin, an mTOR inhibitor. Recently, resistance training has also been shown to affect mitochondrial dynamics, which is coupled with mitochondrial function. In skeletal muscle, mitochondria dynamically change their morphology through repeated fusion and fission, which may be key for controlling the quality of skeletal muscle. However, how the mechanisms of mitochondrial dynamics function during hypertrophy in skeletal muscle remains unclear. The aim of this study was to examine the impact of mTOR inhibition on mitochondrial dynamics during skeletal muscle hypertrophy. Consistent with previous studies, functional overload by synergist (gastrocnemius and soleus) ablation‐induced progressive hypertrophy (increase in protein synthesis and fiber cross‐sectional area) of the plantaris muscle was observed in mice. Moreover, these hypertrophic responses were significantly inhibited by rapamycin administration. Fourteen days of functional overload increased levels of MFN2 and OPA1, which regulate mitochondrial fusion, whereas this enhancement was inhibited by rapamycin administration. Additionally, overload decreased the levels of DRP1, which regulates mitochondrial fission and oxidative phosphorylation, regardless of rapamycin administration. These observations suggest that the relative reduction in mitochondrial function or content is complemented by enhancement of mitochondrial fusion and that this complementary response may be regulated by mTORC1.

## Graphical abstract

Overload‐induced muscle hypertrophy decreased mitochondrial oxidative phosphorylation protein expression and increased fusion‐related protein expression, where the latter may be regulated by mTORC1. These findings suggest that skeletal muscle energy production capacity is maintained by promoting mitochondrial fusion rather than mitochondrial content or function and that mTORC1 is involved as a complex for this adaptation during hypertrophy.

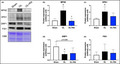

## 1 INTRODUCTION

Skeletal muscle is a highly plastic tissue, and muscle quantity and quality can significantly change when stimulated by exercise and nutrition or when unloaded by inactivity. Skeletal muscle mass is defined by the balance of muscle protein synthesis and degradation, and when synthesis exceeds degradation, skeletal muscle mass increases (Miyazaki & Esser, 2009). Resistance training (RT) and proper nutrition are known to promote muscle protein synthesis and fiber hypertrophy (Damas et al., 2016; Dickinson et al., 2011; Snijders et al., 2016). In particular, the protein kinase mechanistic/mammalian target of rapamycin (mTOR) is well known to play an important role in promoting muscle protein synthesis and subsequent hypertrophy in skeletal muscle (Bodine et al., 2001; Rommel et al., 2001). Therefore, activation of mTOR is one of the important biological responses in RT‐induced skeletal muscle hypertrophy.

mTOR is a common molecule in two functionally distinct multiprotein signaling complexes called mTORC1 and mTORC2. In general, mTORC1‐dependent but not mTORC2‐dependent signaling events are inhibited by the allosteric mTOR inhibitor rapamycin. mTORC1 enhances protein synthesis rates by phosphorylating two downstream signaling proteins, eukaryotic initiation factor 4E‐binding protein 1 (4EBP1) and p70 ribosomal S6 kinase 1 (p70S6K), thereby promoting ribosomal translation efficiency. Collectively, the rapamycin‐sensitive mTOR‐, or mTORC1‐dependent signaling pathways have an essential role in promoting protein synthesis and subsequent skeletal muscle growth and hypertrophy.

Resistance training (RT) is known to increase skeletal muscle hypertrophy via activation of mTORC1, while simultaneously improving mitochondrial function and biogenesis. Twelve weeks of RT has been shown to significantly increase the expression of mitochondrial respiratory chain complex proteins in human skeletal muscle (Porter et al., 2015). In a rodent study, four weeks of RT with electrical stimulation resulted in a significant increase in protein expression of peroxisome proliferator‐activated receptor γ coactivator‐1α (PGC‐1α), a protein involved in mitochondrial biogenesis (Kitaoka et al., 2016; Takegaki, Ogasawara, et al., 2019). Therefore, RT may increase mitochondrial function and biogenesis. In skeletal muscle, mitochondria maintain their quality by forming dynamic networks that are constantly remodeling through fusion and fission. These processes, termed mitochondrial dynamics, are important for mitochondrial quality control. Mitochondrial fusion is regulated by mitofusin proteins (MFN1 and MFN2) and optic atrophy 1 (OPA1), which localize to the mitochondrial outer and inner membranes, respectively (Formosa & Ryan, 2016; MacVicar & Langer, 2016). Mitochondrial fission is mediated by dynamin‐related protein 1 (DRP1), which is localized in the cytoplasm and translocated to the outer membranes when phosphorylated, and mitochondrial fission 1 protein (FIS1) (Romanello et al., 2010). Dysfunction of mitochondrial dynamics can lead to reduced mitochondrial ATP production capacity and increased apoptosis (Bell et al., 2019; Kim et al., 2013). Therefore, mitochondrial dynamics may play an important role in controlling the contraction of skeletal muscle.

In recent years, RT has been shown to alter mitochondrial dynamics. Chronic muscle contraction using electrical stimulation can significantly increase the expression of mitochondrial fusion proteins in rat skeletal muscle (Kitaoka et al., ,,^2015^, ^2016^). Moreover, RT using a similar protocol significantly increased expression of mitochondrial fission proteins (Takegaki, Ogasawara, et al., 2019). Furthermore, a ladder‐climbing exercise with rats, a mimetic model of RT, showed that protein expression of MFN2, FIS1, and DRP1 was significantly increased (Lee et al., 2018). However, the direct mechanism by which RT alters mitochondrial dynamics, and how the muscle mass regulator mTORC1 is involved in skeletal muscle mitochondrial dynamics are not clear. Therefore, the purpose of this study was to examine the influence of mTORC1 on mitochondrial dynamics during skeletal muscle hypertrophy.

## 2 METHODS

### 2.1 Ethics approval

All experimental procedures performed in this study were approved by the Institutional Animal Experiment Committee of the University of Tsukuba (20‐407).

### 2.2 Experimental animals

Male Institute of Cancer Research 7‐week‐old mice (Tokyo Laboratory Animals Science Co, Tokyo, Japan) were purchased from Charles River Laboratories Japan, Inc (Kanagawa, Japan), and housed at temperature (22°C ± 2°C) and humidity (55%±5%)‐controlled holding facilities under a 12‐/12‐hr light/dark cycle and with *ad libitum* access to food and water. The animals were divided into three groups: sham‐operated control mice (Sham, n = 5), synergist ablation‐treated mice (OL, n = 7), and synergist ablation combined with rapamycin‐administrated mice (OL+RA, n = 7).

### 2.3 Synergist ablation surgery

We performed synergist ablation surgeries under anesthesia with isoflurane (2.0%–3.0% isoflurane in air) inhalation as previously described (McCarthy et al., 2011; Miyazaki et al., 2011). This *in vivo* model induces hypertrophy of the plantaris muscle through mechanical overload, resulting from the surgical removal of synergist muscles (gastrocnemius and soleus), for 14 days starting immediately after the synergist ablation surgery. After 14 days, the mice were euthanized by cervical dislocation, and the plantaris muscle was excised, weighed, quickly frozen in liquid nitrogen or liquid nitrogen‐cooled isopentane, and stored at −80°C.

### 2.4 Rapamycin administration

The mTOR inhibitor rapamycin was purchased from Chem Scene LLC (Monmouth Junction, NJ, USA) and was dissolved in dimethyl sulfoxide (DMSO) to generate a 10 µg µl^−1^ stock solution. The appropriate volume of the stock solution needed to inject mice (2.5 mg/kg body weight) was dissolved in 200 µl phosphate‐buffered saline (PBS). For the vehicle control condition, mice were injected with an equivalent amount of DMSO dissolved in 200 µl of PBS. Immediately following the synergist ablation surgeries, vehicle or rapamycin solutions were administrated via intraperitoneal injections; injections were repeated every 24 hr for up to 14 days (Goodman, Frey, et al., 2011; Goodman, Mabrey, et al., 2011).

### 2.5 Western blotting

Isolated plantaris muscles were frozen immediately in liquid nitrogen, and total muscle protein was extracted in lysis buffer containing 50 mM of HEPES (pH: 7.6), 150 mM NaCl, 10 mM EDTA, 10 mM Na_4_P_2_O_7_, 10 mM NaF, 2 mM Na_3_VO_4_, 1% (v/v) NP‐40, 1% (v/v) Na‐deoxycholate, 0.2% (w/v) sodium dodecyl sulfate (SDS), and 1% (v/v) of a complete protease inhibitor cocktail. Protein concentrations were measured using a Protein Assay Bicinchoninate Kit (Nacalai Tesque Inc, Kyoto, Japan). Prior to SDS–polyacrylamide gel electrophoresis (PAGE), an aliquot of the extracted protein solution was mixed with an equal volume of sample loading buffer containing 1% (v/v) 2‐mercaptoethanol, 4% (w/v) SDS, 125 mM Tris–HCl (pH: 6.8), 10% (w/v) sucrose, and 0.01% (w/v) bromophenol blue. Five micrograms of protein was separated in an SDS–PAGE and electrically transferred to an Immuno‐Blot PVDF membrane (Bio‐Rad Laboratories, Hercules, CA, USA). The blot was blocked using Blocking One (Nacalai Tesque Inc.) for 1 hr at room temperature and incubated with primary antibodies overnight at 4°C in Tris‐buffered saline (TBS) with 0.1% Tween‐20. After overnight incubation, membranes were incubated with horseradish peroxidase‐conjugated secondary antibody for 60 min at room temperature. Signals were detected using the ImmunoStar Zeta or LD (FUJIFILM Wako Pure Chemical Co, Osaka, Japan), quantified by C‐Digit (LI‐COR Biosciences, Lincoln, Nebraska, USA), and expressed as arbitrary units. Coomassie Brilliant Blue staining was used to verify consistent loading.

### 2.6 Muscle protein synthesis

Muscle protein synthesis was measured using the *in vivo* SUnSET (surface sensing of translation) method as described previously (Goodman, Frey, et al., 2011; Goodman, Mabrey, et al., 2011). Under anesthesia, 0.04 µM puromycin/g body weight (FUJIFILM Wako Pure Chemical Co.) diluted in a 0.02 M PBS stock solution was injected in the mice intraperitoneally. The plantaris muscle was removed 15 min after puromycin administration. Following homogenization, as described above, and centrifugation at 3800× *g* for 3 min at 4°C, the supernatant was collected and processed for Western blotting. A mouse monoclonal antipuromycin antibody (Merck Millipore, Billerica, MA, USA) was used to detect puromycin incorporation, which was determined as the sum of the intensities of all protein bands in the Western blot.

### 2.7 Hematoxylin and eosin staining

Frozen plantaris muscles were cut as 10‐µm sections using a cryostat (ThermoFisher Scientific K.K, Tokyo, Japan). Sections on the slide glasses were soaked in Mayer's hematoxylin solution (FUJIFILM Wako Pure Chemical Co.) for 10 min, washed with warm water, and then incubated with eosin solution (FUJIFILM Wako Pure Chemical Co.) for 30 s. Sections were dehydrated in 100% ethanol and mounted using Fluorescent Mounting Media (SeraCare Life Science Inc, Milford, MA, USA). Muscle fiber cross‐sectional area (CSA) was measured by circling each fiber per muscle using the Fiji image processing package based on the ImageJ Software (Schindelin et al., 2012).

### 2.8 Transmission electron microscopy

Plantaris muscles were prefixed in 2% glutaraldehyde and 2.5% formaldehyde dissolved in 0.1 M phosphate buffer (PB) for 24 hr at 4°C, followed by three 15‐min washes in 0.1 M PB. After washing, the samples were postfixed in 0.1 M PB with 1% osmium tetroxide for 2 hr at 4°C with gentle shaking and then dehydrated in graded series of ethanol (50%, 70%, 80%, 95%, and 100%) at 25°C. After dehydration, they were infiltrated three times with propylene oxide at 25°C for 20 minutes and then embedded on the resin for 48 hr at 60°C. To check the axial accuracy and cross‐sectional quality of the embedded muscle tissue, 1‐mm‐thick sections were made. Afterward, ultrathin sections were made using a diamond knife on an ultramicrotome and attached to a Pioloform‐filmed copper grids. After staining with uranyl acetate and lead citrate, the sections were photographed using a transmission electron microscope (JEM‐1400; JEOL).

### 2.9 Primary antibodies for Western blotting

The following primary antibodies were used for Western blotting: anti‐eIF4E‐binding protein 1 (4EBP1) (#9452; Cell Signaling Technology), anti‐p‐4EBP1 (Thr37/46, #2855S; Cell Signaling Technology), anti‐p70S6K (#9202; Cell Signaling Technology), anti‐p‐p70S6K (Thr389, #9205; Cell Signaling Technology), anti‐p‐p70S6K (Thr421/Ser424, #9204S; Cell Signaling Technology), anti‐rpS6 (#2217; Cell Signaling Technology), anti‐p‐rpS6 (Ser235/236, #4858S; Cell Signaling Technology), anti‐p‐rpS6 (Ser240/244, #5364P; Cell Signaling Technology), antiglycogen synthase kinase 3β (GSK3β) (#9315; Cell Signaling Technology), anti‐p‐GSK3β (#9336; Cell Signaling Technology), antiextracellular signal‐regulated kinase 1/2 (ERK1/2) (#9102; Cell Signaling Technology), anti‐p‐ERK1/2 (#9101; Cell Signaling Technology), anti‐Rheb (#I2217; Santa Cruz Biotechnology), anti‐PRAS40 (#L0517; Santa Cruz Biotechnology), anti‐PGC‐1α (516557; Merck Millipore), antioxidative phosphorylation (OXPHOS) (ab110413; Abcam), anti‐MFN2 (GR219517‐18; Abcam), anti‐OPA1 (6224762; BD Biosciences), anti‐DRP1 (GR284315‐2; Abcam), and anti‐FIS1 (GR1110‐20; Abcam).

### 2.10 Statistical analysis

Data are shown as means +standard deviation (SD) of the means. Student's *t* test was used for comparisons between the two groups. We performed one‐way ANOVA followed by Tukey's post hoc test. The GraphPad Prism 8 software program (GraphPad, Inc.) was used for all statistical calculations, and the significance level was set to *p* < 0.05 for all cases.

## 3 RESULTS

### 3.1 Body and muscle weight

**FIGURE 1:**
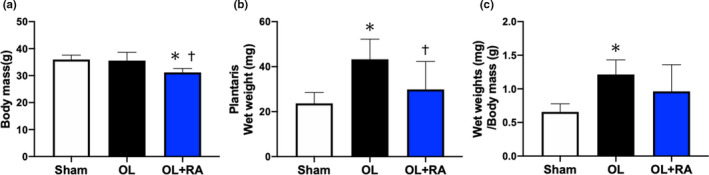
Effects of functional overload (OL) and rapamycin administration on body mass and plantaris wet weight. Body mass (a), plantaris muscle wet weight (b), and plantaris muscle wet weight per body mass (c) were measured after 14 days of OL. Sham, sham operation; OL, functional overload; OL+RA, functional overload combined with rapamycin administration. Data are presented as means +SD. *p < 0.05 versus the Sham group, ^†^ *p* < 0.05 versus the OL group

The changes in body mass and plantaris muscle wet weight were measured following 14 days of functional overload with or without rapamycin administration. Body weight was significantly decreased in the OL+RA group in comparison with both the OL and Sham groups (*p* = 0.0061 vs. Sham, *p* = 0.0060 vs. OL, Figure 1a). Plantaris wet weight and plantaris wet weight/body mass were significantly increased by synergist ablation surgery compared in the Sham group (*p* = 0.0087 vs. Sham, Figure 1b; *p* = 0.0104 vs. Sham, Figure 1c). However, rapamycin administration prevented the increase in plantaris muscle wet weight caused by synergist ablation surgery (*p* = 0.0497 vs. OL+RA, Figure 1b).

### 3.2 Myofibre cross‐sectional area

**FIGURE 2:**
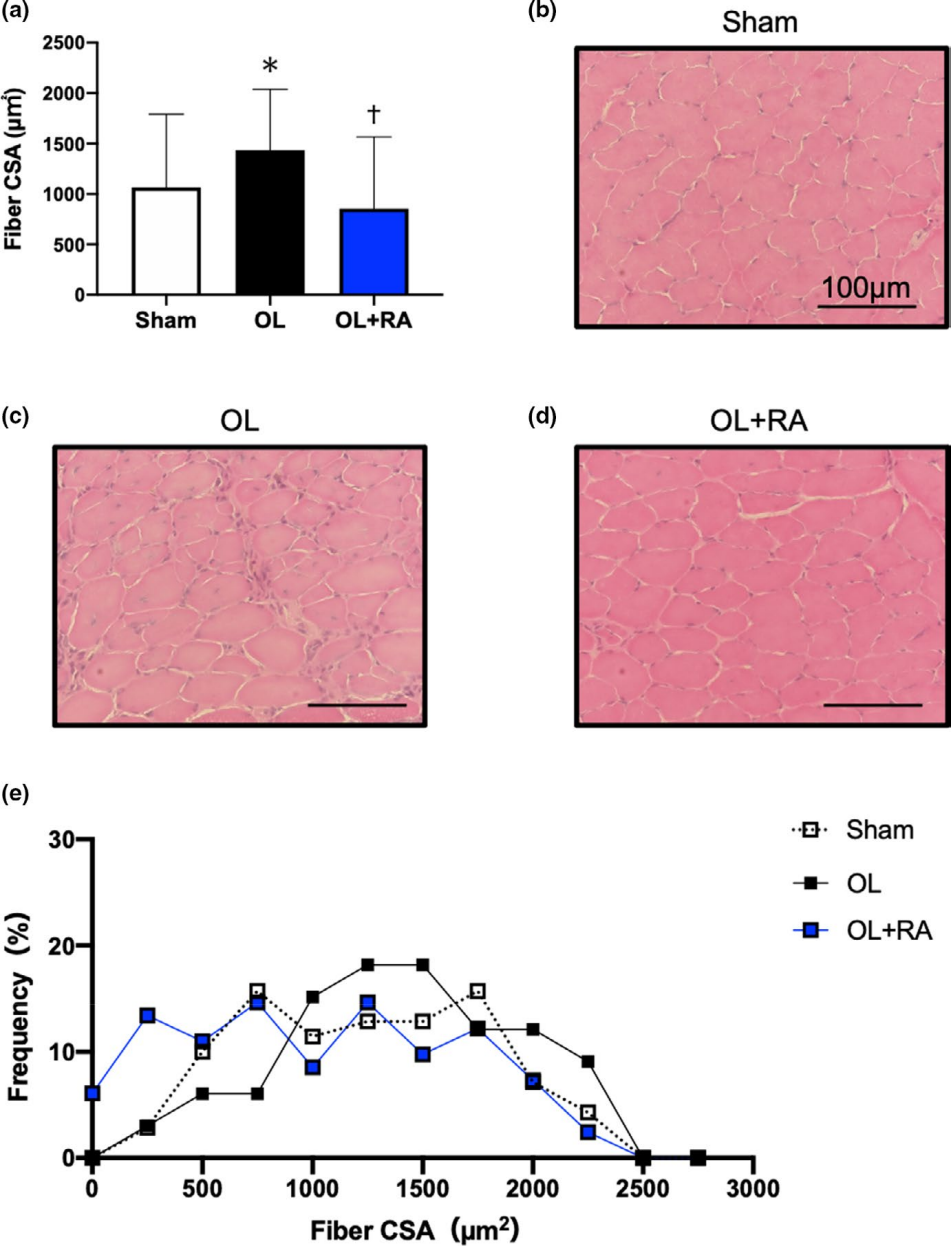
Effects of functional overload and rapamycin administration on muscle fiber CSA (a). Representative microscopic images of Sham (b), OL (c), and OL+RA (d). Distributions of fiber CSA for each experimental group (e). CSA, cross‐sectional area; Sham, sham operation; OL, functional overload; OL+RA, functional overload combined with rapamycin administration. Data are presented as means + SD. *p < 0.05 versus the Sham group, ^†^ *p* < 0.05 versus the OL group

We next investigated the muscle‐hypertrophic effect of each treatment. Myofibre CSA was significantly increased by synergist ablation surgery in the OL group, but not in the OL+RA group (*p* < 0.0001 vs. OL), in comparison with that in the Sham group (*p* = 0.0244 vs. Sham, Figure 2a‐d). These results indicate that administration of rapamycin inhibited skeletal muscle hypertrophy by functional overload.

### 3.3 Skeletal muscle protein synthesis

**FIGURE 3:**
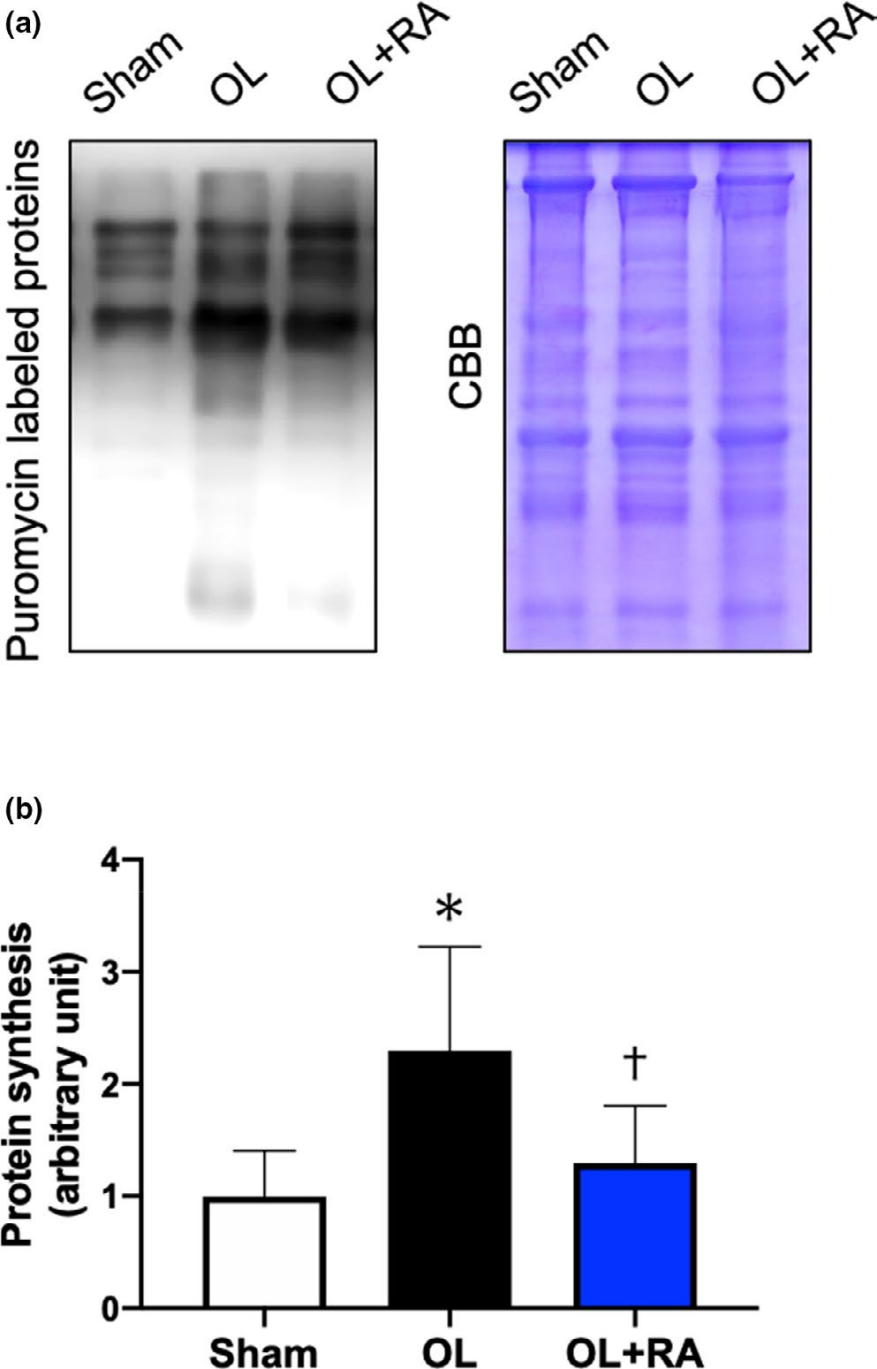
Effects of functional overload and rapamycin administration on muscle protein synthesis. Representative puromycin‐labeled proteins and Coomassie Brilliant Blue‐stained proteins are shown in (a). Puromycin‐labeled proteins in the plantaris muscle after 14 days of functional overload and rapamycin administration (b) were analyzed by Western blotting. Sham, sham operation; OL, functional overload; OL+RA, functional overload combined with rapamycin administration. Data are presented as means + SD. *p < 0.05 versus the Sham group, †*p* < 0.05 versus the OL group

The skeletal muscle protein synthesis rate in plantaris muscle was significantly elevated in the OL group in comparison with that in the Sham group (*p* = 0.0123 vs. Sham). The protein synthesis rate in the OL+RA group was significantly lower than that in the OL group (*p* = 0.0340 vs. OL) and was not significantly different from that in the sham group (Figure 3a‐b).

### 3.4 mTORC1 signaling molecules

**FIGURE 4:**
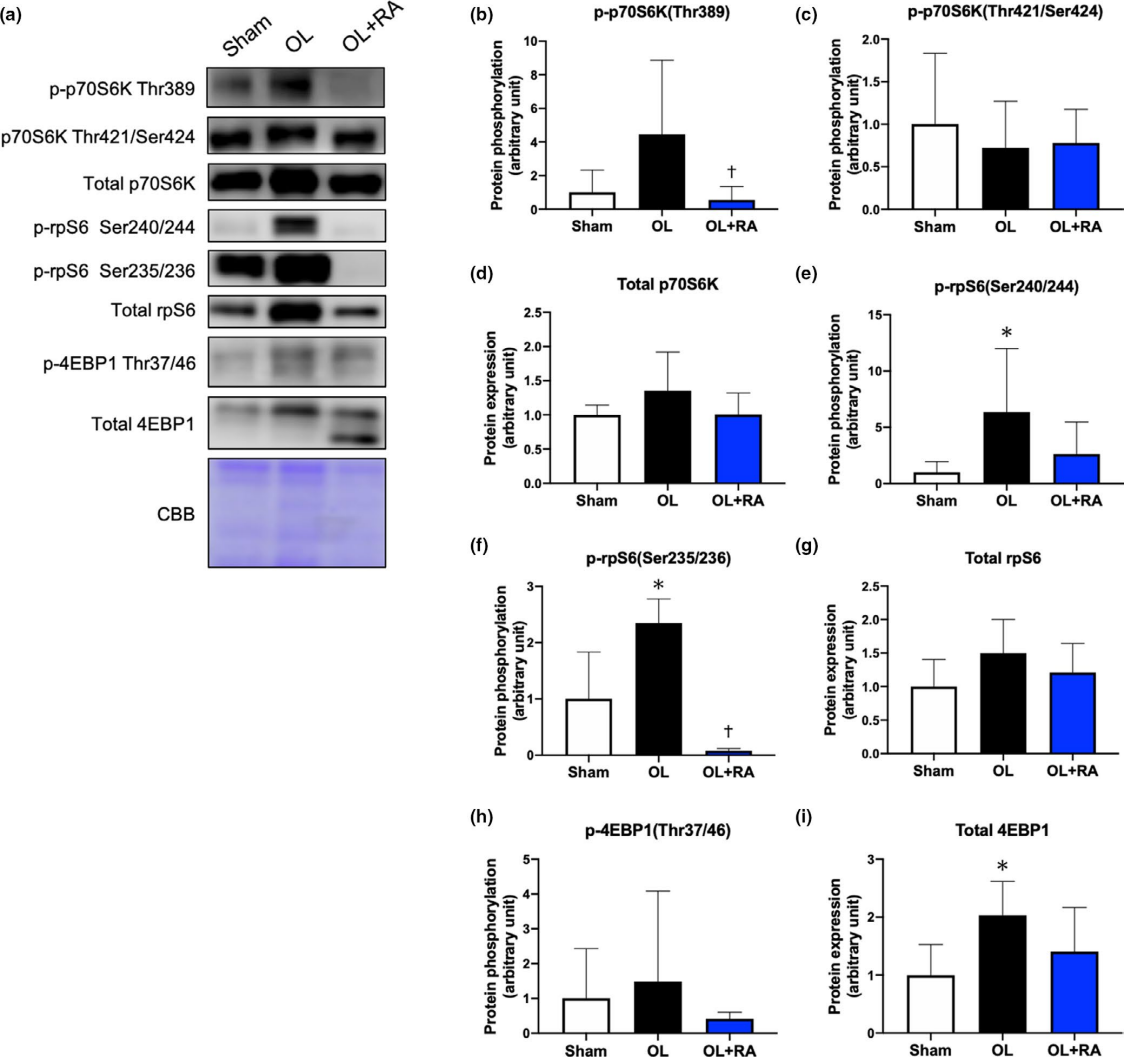
Effects of functional overload and rapamycin administration on the mTORC1 signaling in the plantaris muscle. Representative immunoblots are shown in (a). The protein expressions of phosphorylated p70S6 K (Thr389, b), p70S6 K (Thr421/Ser424, c), total p70S6 K (d), phosphorylated rpS6 (Ser240/244, e), rpS6 (Ser235/236, f), total rpS6 (g), phosphorylated 4EBP1 (Thr37/46, h), and total 4EBP1 (I) in the plantaris muscle after 14 days of functional overload and rapamycin administration were analyzed by Western blotting. Sham, sham operation; OL, functional overload; OL+RA, functional overload combined with rapamycin administration. Data are presented as means + SD. *p < 0.05 versus the Sham group, ^†^ *p* < 0.05 versus the OL group

We investigated the activation of the mTORC1‐dependent signaling pathway, which has an important role in muscle hypertrophy by promoting muscle protein synthesis and ribosomal biogenesis via activation of downstream targets. We measured the phosphorylation levels of p70S6 K, ribosomal protein S6 (rpS6), and 4EBP1 (Figure 4a). Compared with that in the OL group, the expression of phosphorylated p70S6 K (Thr389) was significantly decreased by rapamycin administration (*p* = 0.0485 vs. OL, Figure 4b). However, no significant differences in the phosphorylation states of p70S6 K (The421/Ser424) and the total protein expression of p70S6 K were observed between the OL group and OL+RA group (Figure 4c‐d). Fourteen days of functional overload significantly increased the phosphorylation states of rpS6 (Ser240/244 and Ser235/236, *p* = 0.0042 vs. Sham, Figure 4e; *p* = 0.0007 vs. Sham, Figure 4f) compared with that in the Sham group; however, no significant change in total protein expression was observed between the treated and Sham groups (Figure 4g). Furthermore, the phosphorylation states of rpS6 (Ser235/236) were significantly decreased by rapamycin administration when compared with those in the Sham or OL groups (*p* < 0.0001 vs. Sham or OL, Figure 4f). There was no significant difference in the phosphorylation states of 4EBP1 between the groups, although the total protein expression of 4EBP1 was significantly increased in the OL group compared with that in the Sham group (*p* = 0.0369 vs. Sham, Figure 4h‐i).

### 3.5 Other signaling molecules involved in hypertrophy

**FIGURE 5:**
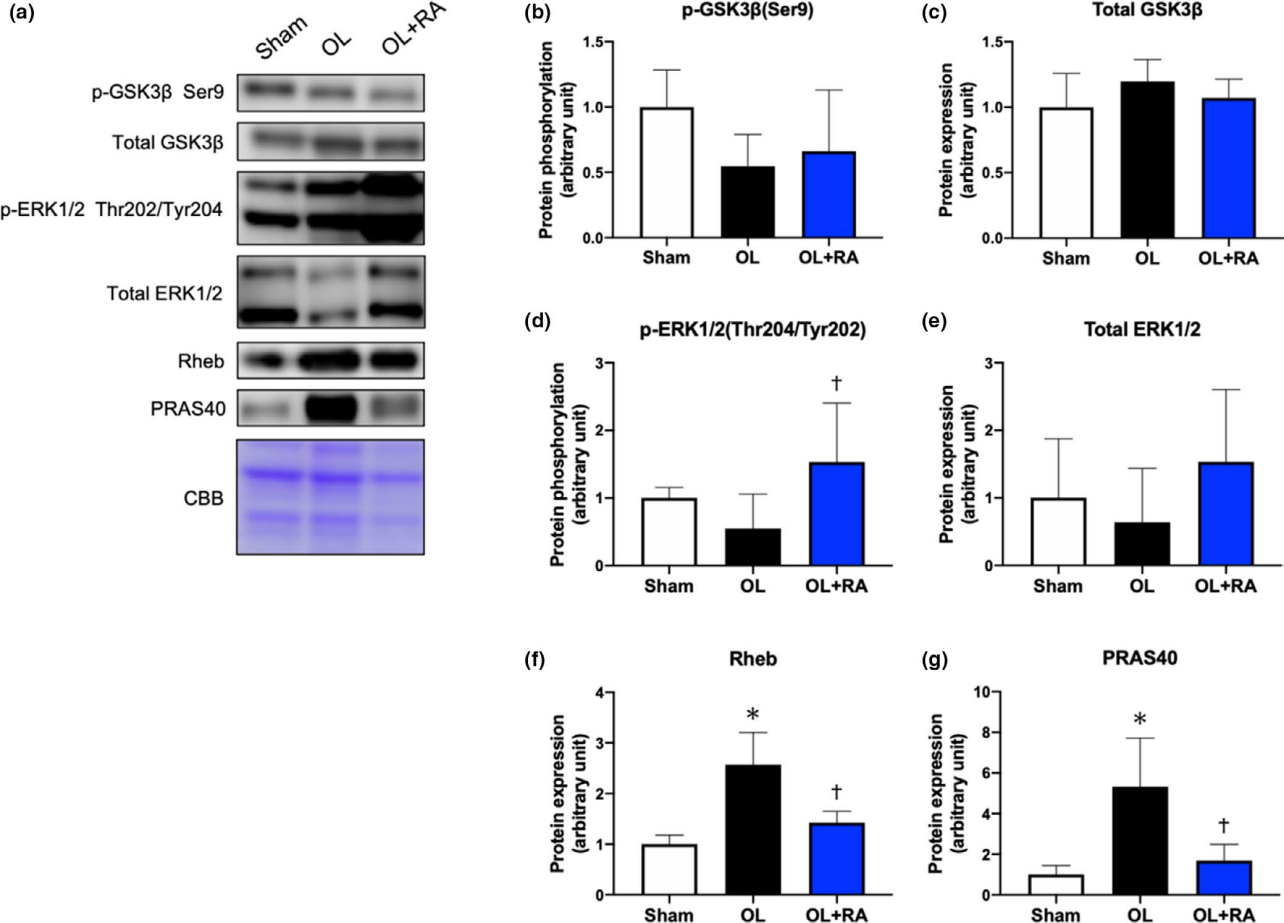
Effects of functional overload and rapamycin administration on the signaling proteins in the plantaris muscle. Representative immunoblots are shown in (a). Protein expressions of phosphorylated GSK3β (Ser9, b), total GSK3β (c), phosphorylated ERK1/2 (Thr202/Tyr204, d), total ERK1/2 (e), Rheb (f), and PRAS40 (g) in the plantaris muscle after 14 days of functional overload and rapamycin administration were analyzed by Western blotting. Sham, sham operation; OL, functional overload; OL+RA, functional overload combined with rapamycin administration. Data are presented as means + SD. *p < 0.05 versus the Sham group, ^†^ *p* < 0.05 versus the OL group

We also measured other signaling molecules including GSK3β, ERK1/2, Rheb, and PRAS40 to clarify their contribution to skeletal muscle cell hypertrophy via either mTORC1‐dependent or mTORC1‐independent mechanisms (Miyazaki et al., 2011; Rommel et al., 2001; Sue et al., 2001). No significant changes in the phosphorylation states and total protein expression of GSK3β were observed between the OL group and OL+RA groups and the Sham group (Figure 5b‐c). Phosphorylation of ERK1/2, which contributes to the activation of p70S6 K, was significantly increased in the OL+RA groups compared with that in the OL group (*p* = 0.0236 vs. OL, Figure 5d). Additionally, no changes were observed in the total protein expression of ERK1/2 between any of the groups (Figure 5e). Rheb, a low molecular weight GTP‐binding protein, is involved in the activation of mTORC1. Functional overload significantly increased the expression of Rheb, in comparison with that of the Sham group (*p* < 0.0001 vs. Sham), but this increase was suppressed by rapamycin administration (*p* = 0.0003 vs. OL, Figure 5f). Similarly, the protein expression level of PRAS40, a subunit of mTORC1, was significantly increased by overload (*p* = 0.0006 vs. Sham) and significantly decreased by rapamycin administration (*p* = 0.0013 vs. OL) in comparison with that in the Sham group (Figure 5g).

### 3.6 Molecules related to mitochondrial biogenesis and OXPHOS

**FIGURE 6:**
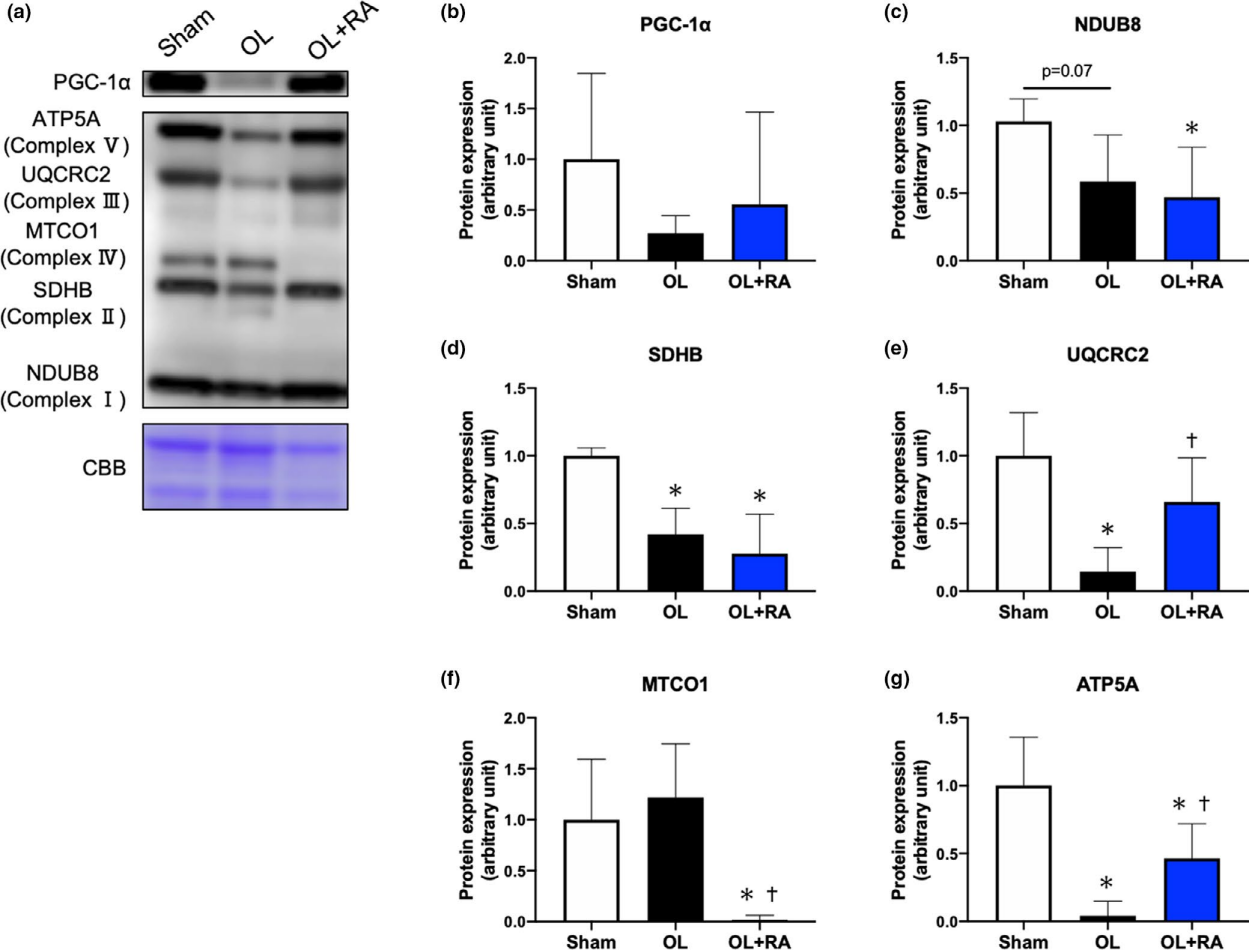
Effects of functional overload and rapamycin administration on the expression of PGC‐1α and proteins involved in mitochondrial oxidative phosphorylation in the plantaris muscle. Representative immunoblots are shown in (a). Expression of PGC‐1α (b), NDUB8 (c), SDHB (d), UQCRC2 (e), MTCO1 (f), and ATP5A (g) in the plantaris muscle after 14 days of functional overload and rapamycin administration was analyzed by Western blotting. Sham, sham operation; OL, functional overload; OL+RA, functional overload combined with rapamycin administration. Data are presented as means + SD. *p < 0.05 versus the Sham group, ^†^ *p* < 0.05 versus the OL group

To evaluate the effect of mTOR inhibition on mitochondrial content, we next investigated the alteration of protein expression levels involved in mitochondrial biogenesis and OXPHOS. The protein expression of PGC‐1α, a master regulator of mitochondrial biogenesis, was decreased by overload, but no significant differences were identified amongst the Sham group and the two treatments (Figure 6b). Among the mitochondrial OXPHOS proteins, as an indicator of mitochondrial content, the protein expression levels of NDUB8 (Complex Ⅰ) were significantly lower in the OL+RA group than those in the Sham group (*p* = 0.0227 vs. Sham, Figure 6c). The expression of SDHB (Complex Ⅱ) was significantly lower in the OL and OL+RA groups than that in the Sham group (*p* = 0.0008 vs. Sham, *p* < 0.0001 vs. Sham, Figure 6d). The expression of ATP5A (Complex Ⅴ) was significantly lower in the OL and OL+RA groups than that in the Sham group (*p* < 0.0001 vs. Sham, *p* = 0.0051 vs. Sham, Figure 6g). Additionally, the expression of ATP5A was significantly higher in the OL+RA group in comparison with the OL group (*p* = 0.0143 vs. OL, Figure 6g). The expression of UQCRC2 (Complex Ⅲ) was significantly decreased in the OL group in comparison with that in the Sham group (*p* = 0.0002 vs. Sham); however, this was significantly increased in the OL+RA group in comparison with that in the OL group (*p* = 0.0084 vs. OL, Figure 6e). Furthermore, the protein expression of MTCO1 (Complex Ⅳ) was not altered by overload, but was significantly decreased by the combination of overload and rapamycin administration compared with that in the Sham and OL group (*p* = 0.0042 vs. Sham, *p* = 0.0003 vs. OL, Figure 6f).

### 3.7 Mitochondrial morphology

**FIGURE 7:**
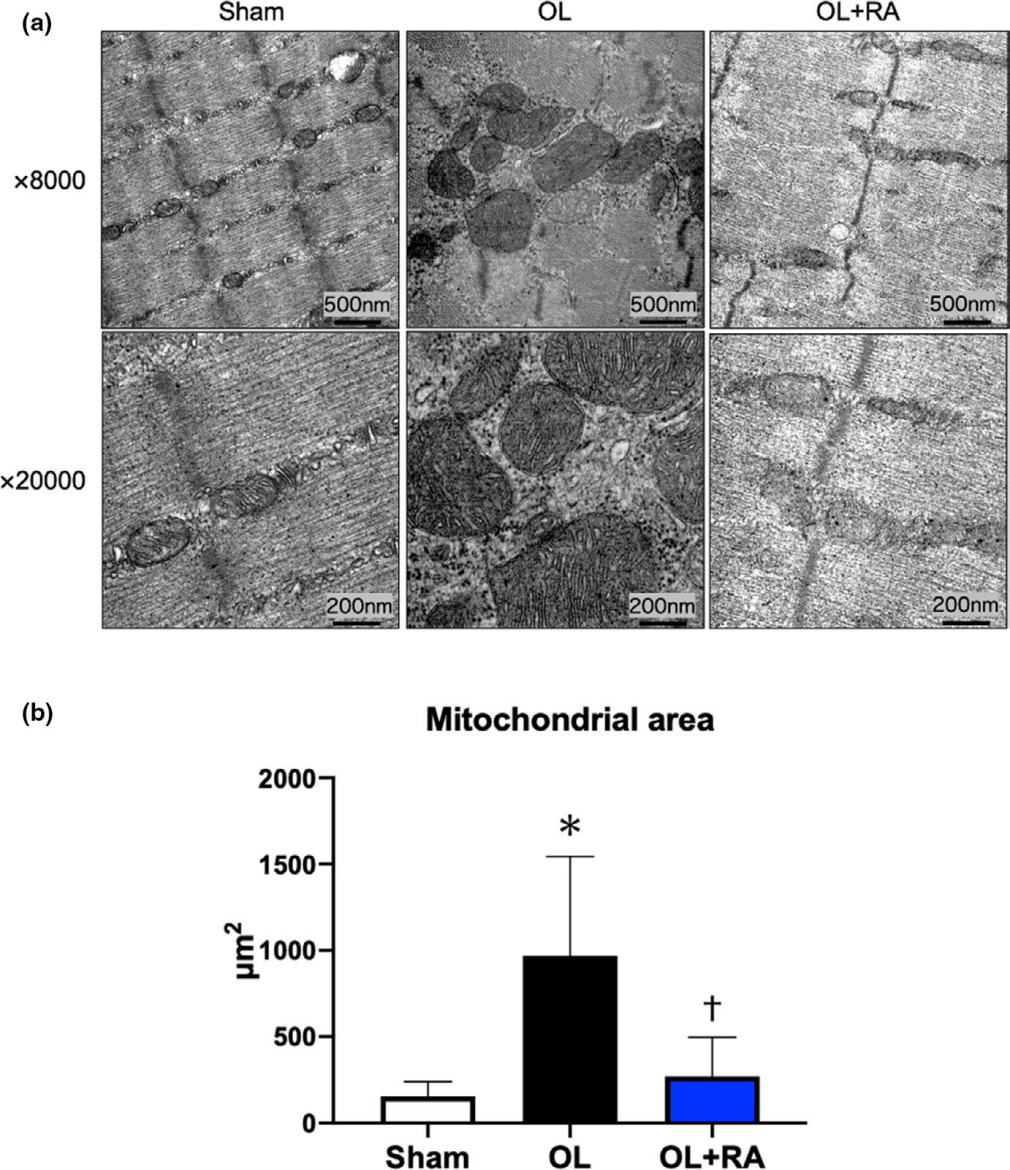
Effects of functional overload and rapamycin administration on the mitochondrial morphology in the plantaris muscle. Representative electron micrographs are shown in (a). Statistical evaluation of mitochondrial area is shown in (b). Sham, sham operation; OL, functional overload; OL+RA, functional overload combined with rapamycin administration. Data are presented as means + SD. *p < 0.05 versus the Sham group, ^†^ *p* < 0.05 versus the OL group

Electron micrographs show that mitochondria in the OL group have an enlarged and highly cristaerized morphology compared with those in the sham and OL+RA groups (Figure 7a). Mitochondrial area was significantly increased in the OL group compared with Sham group (*p* < 0.0001 vs. Sham), but that was significantly lower in the OL+RA group compared with OL group (*p* < 0.0001 vs. OL, Figure 7b).

### 3.8 Mitochondrial dynamics‐related proteins

**FIGURE 8:**
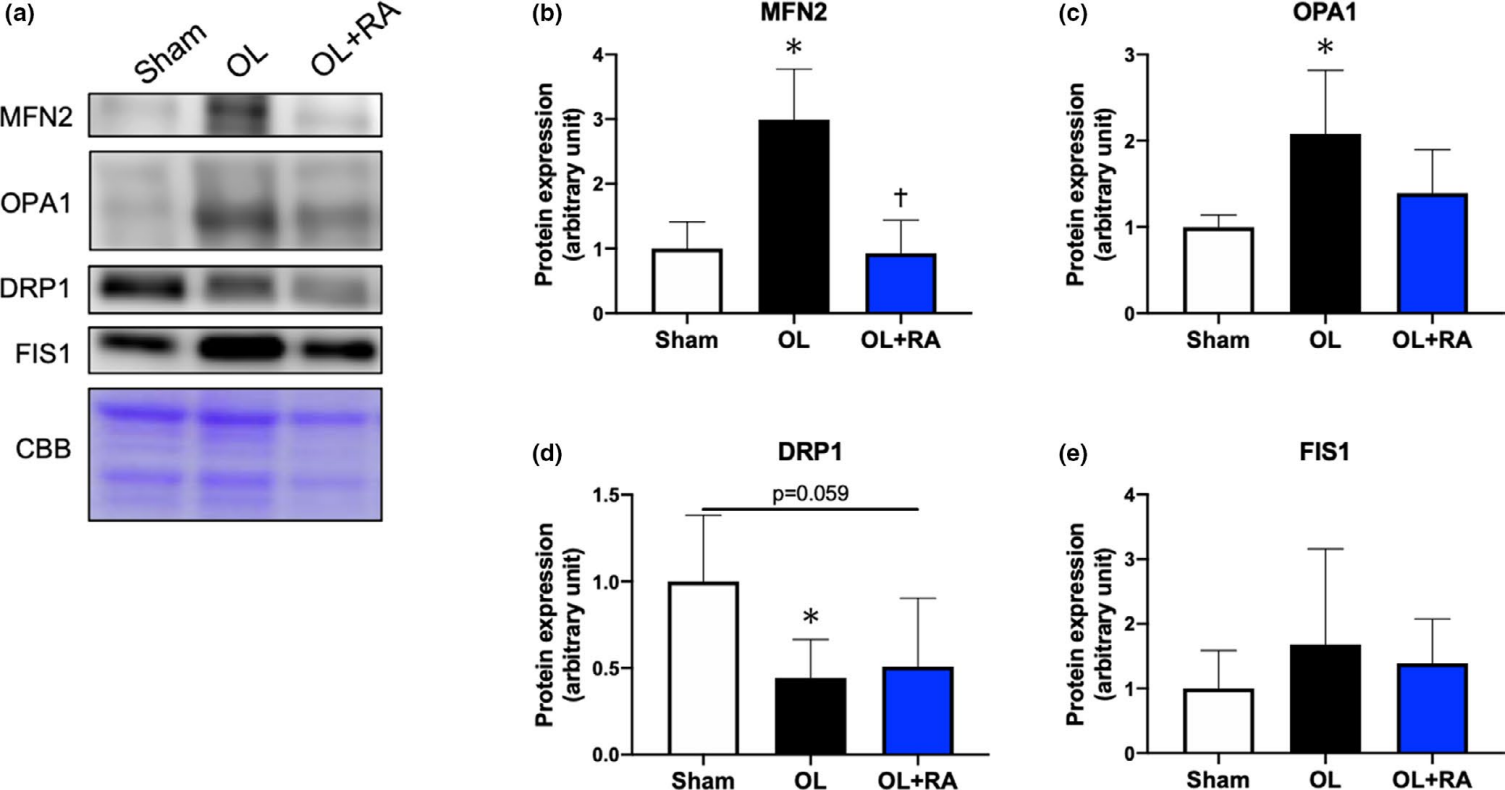
Effects of functional overload and rapamycin administration on the mitochondrial dynamics proteins in the plantaris muscle. Representative immunoblots are shown in (a). Protein expressions of MFN2 (b), OPA1 (c), DRP1 (d), and FIS1 (e) in the plantaris muscle after 14 days of functional overload and rapamycin administration were analyzed by Western blotting. Sham, sham operation; OL, functional overload; OL+RA, functional overload combined with rapamycin administration. Data are presented as means +SD. *p < 0.05 versus the Sham group, ^†^ *p* < 0.05 versus the OL group

Functional overload increased the expression levels of MFN2 in comparison with those in the Sham group (*p* = 0.0001 vs. Sham), but rapamycin administration suppressed these responses (*p* < 0.0001 vs. OL, Figure 8b). Additionally, the expression levels of OPA1 were significantly higher in the OL group compared with the Sham group (*p* = 0.0105 vs. Sham, Figure 8c) but not in the OL+RA group. In contrast, functional overload decreased the expression levels of DRP1 in comparison with that in the Sham group, with a slightly lower decrease in the OL+RA group (*p* = 0.0307 vs. Sham, *p* = 0.0593 vs. Sham, Figure 8d). However, we did not find any significant difference in the expression levels of FIS1 between the groups (Figure 8e).

## 4 DISCUSSION

In this study, we examined the effects of mTORC1 on mitochondrial dynamics during skeletal muscle hypertrophy induced by synergist ablation. Functional overload‐induced promotion of muscle protein synthesis, mTORC1 signaling activation, and muscle hypertrophy, although those were inhibited by rapamycin administration. The main findings of this study are as follows: (1) expression levels of mitochondrial OXPHOS proteins were decreased regardless of functional overload and rapamycin administration; (2) functional overload for 14 days in the absence of rapamycin administration, increased mitochondrial fusion protein expression levels; (3) rapamycin administration inhibited functional overload‐induced muscle hypertrophy as well as expression of mitochondrial fusion‐related proteins.

We first investigated the effects of rapamycin administration on body mass and plantaris wet weight after 14 days of functional overloaded and observed significant body mass loss in the OL+RA group, consistent with previous studies (Bentzinger et al., 2008; Zhang et al., 2019). In addition, functional overload significantly increased plantaris muscle wet weight and CSA, whereas rapamycin administration prevented an increase in muscle wet weight and CSA. Since these results were consistent with previous studies (Miyazaki et al., 2011; Moriya & Miyazaki., 2018), the muscle hypertrophy model adopted in this study was an appropriate experimental protocol. Recent studies demonstrated that rapamycin administration significantly inhibited muscle hypertrophy induced by RT, synergist ablation, and myotenectomy (Goodman, Frey, et al., 2011; Goodman, Mabrey, et al., 2011; Ogasawara et al., 2016; You et al., 2019).

We then investigated the effect of rapamycin administration on activation levels of signaling molecules for muscle hypertrophy by functional overload. In this study, functional overload significantly increased the expression levels of puromycin‐labeled proteins, but not in mice treated with rapamycin. In an attempt to further elucidate the underlying regulation of muscle hypertrophy, we investigated the activation status of molecules in mTORC1 signaling pathways and determined that rapamycin administration inhibited the functional overload‐induced phosphorylation levels of p70S6 K (Thr389), rpS6, and the total protein expression levels of 4EBP1. Since these results were consistent with previous studies (Maruyama et al., 2020; Ogasawara et al., 2017, 2020; Ogasawara & Suginohara., 2018; Takegaki et al., 2017), rapamycin administration may have inhibited mTORC1 signaling activation and subsequent muscle protein synthesis induced by functional overload.

Mitogen‐activated protein kinase signaling has been shown to be involved in protein synthesis associated with muscle contraction (Takegaki, Ogasawara, et al., 2019). We demonstrated that rapamycin administration enhanced the phosphorylation levels of ERK1/2. Previously, expression levels of phosphorylated ERK1/2 were shown to be unaffected by rapamycin (Miyazaki & Takemasa., 2017). The augmentation of ERK1/2 phosphorylation by rapamycin is a new finding from our study. Additionally, we found that protein expression levels of Rheb, a direct activator of mTORC1, and PRAS40, a subunit of mTORC1, were enhanced by functional overload, although they were suppressed by subsequent rapamycin administration. In a human study, colocalization of mTOR and Rheb has been suggested to enhance mRNA translational capacity after resistance exercise (Song et al., 2017). The phosphorylation levels of PRAS40 were significantly increased by functional overload for 14 days (Moriya & Miyazaki., 2018), suggesting that colocalization of mTOR with Rheb and enhancement of total protein expression level of PRAS40 are essential for hypertrophy.

Finally, we examined the effects of mTORC1 inhibition on the expression levels of proteins involved in mitochondrial biogenesis, OXPHOS, and mitochondrial dynamics. We did not observe any significant difference in the protein expression of PGC‐1α between the groups, although the expression levels of OXPHOS proteins were significantly decreased by functional overload regardless of rapamycin administration. Chronic muscle contraction has been demonstrated to enhance expression levels of PGC‐1α and OXPHOS proteins (Kitaoka et al., 2015; Ogasawara et al., 2016; Takegaki, Ogasawara, et al., 2019). While these previous studies employed a model of muscle hypertrophy induced by 4 weeks of muscle contraction, our study employed a model of rapid muscle hypertrophy in 14 days, and we believe that adequate adaptation of mitochondrial biogenesis and mitochondrial content would not occur in this short‐term muscle hypertrophy model. In other words, the increase in mitochondrial content failed to adapt to muscle hypertrophy, and mitochondrial content decreased relatively. However, administration of rapamycin has been suggested to be insufficient to prevent increases in expression of PGC‐1α immediately after endurance exercise or 4 weeks of RT (Ogasawara et al., 2016; Philp et al., 2015). Here, we did not observe any changes in protein expression levels of PGC‐1α with rapamycin administration, suggesting that differences in exercise mode and loading pattern may affect adaptation of PGC‐1α expression. Moreover, the expression of OXPHOS proteins was significantly reduced in both OL and OL+RA groups. Assuming that the reduction in OXPHOS expression in the OL group is a response to rapid muscle hypertrophy over a short‐term period, the reduction in the expression of OXPHOS proteins associated with the suppression of hypertrophy by rapamycin administration suggests that mTORC1 may have an effect on the adaptation of mitochondrial content or function associated with muscle hypertrophy.

We observed enlarged and highly cristaerized mitochondrial morphology in the OL group but not in the Sham or OL+RA groups. In the OL group, disruption of sarcomere structure (including Z‐disk and actomyosin filaments) was observed, whereas this was not as observed in the OL+RA group. Conceivably, enlarged and highly cristaerized mitochondria in the OL group may contribute to promote energy production required for regeneration of muscle structure disrupted by overload with synergist ablation. We assume the disruption of sarcomere structure is at least partially mTORC1‐dependent since rapamycin seems to partially suppress the disruption even with the same overload. This result is demonstrated by several TEM images, but there still remains possibility that mitochondria have swollen in one axis but shrunk in another axis owing to cytoskeletal changes during overload period. We found that the expression levels of MFN2 and OPA1 were increased in the OL group, but not in the OL+RA group. However, the expression level of DRP1 was significantly decreased in the OL group and tended to decrease in the OL+RA group. Previous studies demonstrated that 4 weeks of muscle contraction enhanced the expression levels of MFN1/2 and OPA1 (Kitaoka et al., ,,^2015^, ^2016^). Additionally, 18 sessions of muscle contraction were reported to significantly increase not only the protein expression levels of MFN2 and OPA1 but also those of DRP1 and FIS1 (Takegaki, Ogasawara, et al., 2019). Four weeks or three months of endurance training significantly increased the expression levels of FIS1 in rat skeletal muscle, although those of MFN1/2 were decreased by endurance training (Feng et al., 2013; Marton et al., 2015). In human studies, 2 weeks, with 3 to 4 days per week of high‐intensity interval training, or 12 weeks of moderate‐intensity cycling exercise training increased the expression levels of both MFN1/2, OPA1, and FIS1 (Konopka et al., 2014; Perry et al., 2010). These previous studies indicated that the adaptation of mitochondrial fusion and fission to exercise training is greatly influenced by differences in exercise mode. Our findings indicate that the increased expression of mitochondrial fusion‐related proteins and decreased expression of fission‐related proteins are functional overload‐specific adaptations. Furthermore, in this study, rapamycin administration prevented the increase in the protein expression levels of MFN2 and OPA1 with 14 days of functional overload. Civiletto et al reported that overexpression of OPA1 enhanced the expression of OXPHOS proteins in mice with muscle‐specific knockout of COX15, a regulator of the oxidoreductase activity of complex Ⅳ (Civiletto et al., 2015). Given that rapamycin administration significantly inhibited muscle hypertrophy, the muscle‐hypertrophic response may be accompanied by the activation of mitochondrial fusion, and we postulated that this was a compensatory response to the decreased expression of OXPHOS proteins by 14 days of functional overload. However, suppression of enhanced expression of mitochondrial fusion‐related proteins by rapamycin administration may be a response to mTORC1 regulation, but may also be the result of declined muscle‐hypertrophic response and requires further investigation of the exact mechanism. Morita et al reported that mTORC1 inhibition against mouse embryonic fibroblasts suppressed DRP1 Ser616 phosphorylation (Morita et al., 2018). Similarly, in the present study, we found that DRP1 expression tended to decrease in the OL+RA group. Therefore, mTORC1 may regulate mitochondrial fission. Interestingly, we found that DRP1 expression was also decreased in the OL group. A previous study reported that chronic muscle contractile activity over 7 days significantly decreased DRP1 expression (Iqbal et al., 2013). However, a previous study reported that mTORC1 activation by deletion of TSC2 promoted DRP1 Ser616 phosphorylation (Morita et al., ). These findings suggest that mTORC1, which is activated during muscle hypertrophy, inhibits mitochondrial fission rather that enhances that through a mechanism specific to chronic mechanical overload.

In summary, we suggest that promotion of mitochondrial fusion during muscle hypertrophy may be inhibited by mTORC1 inhibition. In future research, to determine the relationship between mitochondrial dynamics and skeletal muscle hypertrophy, the effects of changes in mitochondrial dynamics on muscle‐hypertrophic response following RT should be investigated.

## CONFLICT OF INTEREST

The authors declare that there are no conflicts of interest.

## AUTHOR CONTRIBUTIONS

All authors conceived and designed the project; K.U. and H.H. performed the experiments; K.U. and T.S. analyzed the data; K.U. wrote the paper; K.U. and T.T. made manuscript revisions. All authors read and approved the final manuscript.
